# Latent Profile Analysis of Positive Solitude During the Recurrent Outbreak of COVID-19

**DOI:** 10.3389/fpubh.2022.872128

**Published:** 2022-05-31

**Authors:** Zhijun Yu, Baojuan Ye, Yong Hu, Qiang Yang

**Affiliations:** ^1^Center of Mental Health Education and Research, School of Psychology, Jiangxi Normal University, Nanchang, China; ^2^School of Foreign Languages, Post-doctoral Research Station of Psychology, Jiangxi Normal University, Nanchang, China; ^3^School of Education, Jiangxi Normal University, Nanchang, China

**Keywords:** positive solitude, latent profile analysis, positive and negative affect, COVID-19, Chinese adults

## Abstract

The current study aimed to identify latent profiles of positive solitude during the recurrent outbreak of COVID-19 among Chinese adults. A total of 902 adults from China completed the questionnaires. We found five different profiles of positive solitude: low positive solitude group, medium-low positive solitude group, quietness positive solitude group, medium-high positive solitude group, and high positive solitude group. Positive and negative affect were significantly different from the five profiles. In addition, gender had different effects on different positive solitude profiles. The results of the study provide a new perspective to understand the positive solitude of Chinese adults during the recurrent outbreak of COVID-19 by using the people-centered approach.

## Introduction

Since 2020, the novel coronavirus disease COVID-19 has become a global epidemic. Due to the difficulties of epidemic prevention, COVID-19 has repeatedly broken out in countries around the world. In order to avoid the further spread of COVID-19, the Chinese government has taken many preventive measures, such as succeeding social isolation, limiting traffic and travel, controlling social distance, and studying or working online. And thus individuals' scope of activities narrowed and social activities decreased in this situation. Solitude became the norm for most Chinese people in the COVID-19 period. Some studies have shown that quarantine can affect individuals' physical and mental health (1–3), which varies from person to person (4). While in quarantine, some people may feel depression and anxiety, however, others can remain optimistic, and view it as a kind of positive experience (4). Therefore, solitude does not always bring people anxiety and fear. Even in isolation during the epidemic, some people can remain calm and have a positive experience of privacy, which is commonly referred to as positive solitude.

Positive solitude refers to individuals' ability to positively enjoy alone time without meaningful interaction with others (5). In the whole process of life, individuals have more or less needs for solitude to relieve social pressure, reflect on themselves and conduct emotional renewal (6–10). Compared to Western cultures, Eastern cultures are more concerned with solitude (11, 12), especially Chinese Confucianism and Taoism, which both advocate solitude. This is because in the traditional Chinese cultural concept, solitude is the best way to reflect one's ability and exercise one's psychological qualities as well. Therefore, in the context of Chinese culture, solitude is deemed to be a method of self-cultivation and has been widely implemented in educational practice. During the recurrent outbreaks of COVID-19, individuals face the risk of being quarantined at any time. Studies have shown that positive solitude reduces bad behavior and benefits mental health (10, 13). Thus, positive solitude may have positive and constructive implications for individuals during COVID-19. Given the importance of positive solitude, it is necessary to investigate individuals' positive solitude during the recurrent outbreak of COVID-19.

### Positive Solitude, Solitude and Loneliness

The concept of solitude was first introduced in 1959 by the sociologist Goffman, and Goffman argued that when individuals are in solitude, they feel the joy of being liberated (14). However, scholars tend to associate solitude with loneliness in some early studies (15, 16). From an evolutionary psychological point of view, solitude is not conducive to the survival of the weak in the environment where the fittest survive. The way humans choose to live in groups and engage in various altruistic behaviors is to improve the fitness of the whole race and thus provide a better chance of passing on their genes (17, 18). Therefore, individuals should avoid solitude as much as possible to increase the possibility of survival (19). In addition, the negative aspect of solitude is mainly loneliness, which many psychologists have studied since loneliness may cause some emotional disorders and affect individuals' mental health (20). As a result, early scholars often focused on the negative aspects of solitude, while the positive aspects of solitude were often ignored.

Nonetheless, some researchers have proposed that solitude and loneliness are two different concepts (21, 22). The concept of “loneliness” first appeared in the field of psychiatry and referred to the impairment of interpersonal communication and emotional expression. From a medical point of view, loneliness is caused by abnormalities in social functioning and communication. In 1973, Weiss published an article on loneliness, which formally introduced the concept of loneliness into the field of psychology (23). Loneliness refers to a painful psychological experience that individuals experience when they feel that their expectations cannot be met in current social interaction (24). However, solitude does not always have to be painful, and it can also be seen as a positive state of being sought rather than avoided (8). Winnicott is the first to regard solitude as a basic positive development ability of individuals, and emphasized the importance of individuality and privacy (25). In Maslow's opinion, the need for solitude is a feature of self-actualizer, who are willing to and actively seeks opportunities to be alone (26). With the reinterpretation of solitude, researchers gradually break away from the prejudice against solitude and turn to the study of its possible positive effects.

Positive solitude was first proposed by Ost Mor in 2020 (27), and Palgi first clearly distinguished positive solitude as an independent solitary capacity from loneliness and solitude in a quantitative way (5). Positive solitude is individuals' purposeful and active choice accompanied by positive affect experience (28). It does not only help individuals to introspect, self-regulate and enhance creativity (27–29), but also plays an important and active role in the mental health of individuals. Moreover, positive solitude may also have great importance during the recurrent outbreak of COVID-19.

### Person-Centered Approach to Positive Solitude

There are qualitative differences among individuals in positive solitude, which can be divided into multiple categories. For example, a qualitative study conducted by Ost Mor et al. investigated 124 participants through interviews, and divided them into seven categories according to different characteristics of individuals' positive solitude (27). Although this study revealed the heterogeneity of positive solitude, the results of qualitative research were difficult to generalize and had limited representativeness (30). In addition, most studies on positive solitude are variable-centered (5, 31). This variable-centered approach mainly uses the total score of the variable or the score of each dimension of the variable. The basic assumption is that there is homogeneity among subtypes, ignoring qualitative differences between individuals. Latent profile analysis (LPA) is a person-centered approach to classify individuals based on response patterns on a set of items, which can ensure the maximum difference between potential categories and the minimum difference within subtypes (32, 33). Meanwhile, it can judge the proportion of different subtypes in the whole population according to the responses of subjects in each item, so as to capture the group inequality that cannot be observed in variable-centered studies (34). Therefore, this study intends to use the method of latent profile analysis to explore latent profiles of positive solitude among Chinese adults. And this was the first goal of the study.

The second goal of this study was to explore the effect of gender on positive solitude. In the previous variable-centered studies, there are some debates on the impact of gender on positive solitude. Some studies have found no difference between males and females in their preference for solitude (35–37), whereas other studies found a significant difference (38). Wong and Csikszentmihalyi assumed that girls, because of their higher affiliation motivation, might have a more negative response for solitude than boys, however, their results did not support the hypothesis (39). Different patterns of positive solitude in different individuals may lead to this mixed pattern. Therefore, this study will use LPA to more accurately explore the impact of gender on positive solitude patterns among Chinese adults during the recurrent outbreak of COVID-19. Maes et al. tested about 1,800 adolescents' attitudes toward solitude. They analyzed the results using a people-centered approach, which showed higher scores for females than males in affinity for solitude (40). Therefore, we hypothesized that females have a higher capacity for positive solitude than males.

Finally, our third goal was to examine the association between positive solitude and affect. From the perspective of valence, affect can be divided into positive affect and negative affect (41). Modern medical research results show that the change of affect can directly affect a variety of physiological activities in the human body (42). Positive affect can reduce personal stress feelings, thus improving individuals' health and quality of life (43). Negative affect will influence many aspects of individuals' life, bringing a series of severe consequences, for example, negative affect will affect physiological function, interfere with the immune system, and also induce various psychosomatic and mental diseases (43, 44). Some research showed that positive solitude was accompanied by positive affect (5, 45). The research further found that groups with “positive solitude experience” were characterized by positive affect, and almost exhibited no negative affect (45). Palgi et al. (5) also found that positive solitude was positively correlated with positive affect. Therefore, this study will explore the relationship between latent categories of positive solitude and affect from a people-centered perspective. We hypothesized that, in general, adults with higher levels of positive solitude would have higher positive affect and lower negative affect.

### The Present Research

The first purpose of this study was to explore the latent classes of positive solitude by using latent profile analysis. The second objective was to examine the differences in gender between different latent types of positive solitude. A final objective was to investigate differences in positive and negative affect among different latent classes of positive solitude.

## Methods

### Participants

A sample of 902 adults (367 males and 535 females) from China completed the questionnaires. Among them, 33.92% (*n* = 306) were aged 18 to 24, 31.71% (*n* = 286) were aged 25 to 30, 27.16% (*n* = 245) were aged 31 to 40 and 7.21% (*n* = 65) were aged 41 to 60. In addition, among the 902 participants, 7.21% (*n* = 65) have high school degree or below, 22.51% (*n* = 203) have associate degree, 65.30% (*n* = 589) have bachelor degree, and 4.99% (*n* = 45) have master degree or above.

### Procedure

Nine hundred and two participants from China completed the survey. Specifically, this research publishes recruitment information on the internet, and interested participants can participate in the research. Participants completed a survey anonymously to collect information on gender, age group, positive solitude, and positive or negative affect. The survey was hosted on questionnaire web (Shanghai Zhongyan International Science and Technology, Shanghai, China; https://www.wenjuan.com/). Before completing the questionnaire, all participants read and signed the informed consent form. In this study, all responses were anonymous. There was no compensation for participating in this study, and the participants participated entirely voluntarily. The study was approved by the ethics committee of the First Author's University.

### Measures

#### Positive Solitude Scale

The nine-item and one-dimensional Positive Solitude Scale (PS) was used to measure the positive solitude ability (5). Individuals rated each item (e.g., “*When I find time for myself, I succeed better at making future plans*”) on a five-point scale ranging from 1 (*never*) to 5 (*always*). Higher total scores indicated higher positive solitude. This study used a forward and backward translation technique to translate the Positive Solitude Scale into Chinese (46). First, five graduate students majoring in psychometrics translated the scale according to the Chinese cultural background and language expression habits respectively. Then, the Chinese version was back-translated into English by two bilingual individuals. Finally, a psychometrics expert compared the back-translate English version with the original questionnaire, and some Chinese expressions were altered without changing the items' meaning. In the present study, exploratory factor analysis revealed that the Chinese version of Positive Solitude Scale included one dimension, which explained the total variance of 44.94%. The confirmatory factor analysis showed that the inventory had good construct validity (χ^2^/df = 3.02, CFI = 0.96, TLI = 0.94, SRMR = 0.03, RMSEA = 0.07) (47, 48). The Cronbach's α coefficient of the Chinese version of Positive Solitude Scale was 0.85. In addition, the Chinese version of the Positive Solitude Scale was significantly positively correlated with positive affect (*r* = 0.52, *p* < 0.001), and negatively correlated with negative affect (*r* = −0.11, *p* < 0.01). Therefore, the Chinese version of Positive Solitude Scale has good reliability and validity, and hence can be used as a tool to measure positive solitude of Chinese adults. The contents of the questionnaire for the nine items are shown in Table 1.

**Table 1:** Contents of the nine-item questionnaire.

**No**.	**Item**
1	When I find time for myself, I succeed better at making future plans.
2	I like carving out time to enjoy being by myself in a pleasant place/environment.
3	I enjoy carving out time for myself to look outside my house or gaze at the scenery.
4	When I find time for myself, I feel focused and enable to achieve my best results.
5	When I am by myself, I can achieve the high level of focus that I need.
6	When I am stressed, having time by myself helps me clear my mind.
7	Time for myself gives my life more meaning.
8	Time for myself enhances my creativity.
9	Finding time for myself contributes to my quality of life.

#### Positive and Negative Affect Scale

The Chinese version (49) of Positive and Negative Affect Scale (PANAS) (50) was used to measure positive and negative affect. The scale consists of 20 items and includes two dimensions, of which 10 items are positive affect (e.g., “*interested*”) and 10 items are negative affect (e.g., “*upset*”). Each item was rated on a five-point scale (1 = *very slightly* to 5 = *not at all*), with higher scores of positive/negative affect subscale representing higher positive/negative affect. In the present study, Cronbach's α were 0.91 and 0.94 for the positive affect subscale and negative affect subscale.

### Analytical Approach

Firstly, Mplus 7.0 is used to analyze the latent profile of college students' positive solitude. By referring to Nylund et al. (51), the two classes model was used as a reference point. We gradually increase the number of model classes for parameter estimation, and we compare these models to find the best model. Some indexes were employed to determine model fit (52–55), including Akaike Information Criterion (AIC) (56), Bayesian Information Criterion (BIC) (57), sample-size-adjusted BIC (aBIC) (58), Entropy (59), Lo-Mendell-Rubin likelihood ratio test (LMR-LRT) (60), and Bootstrap likelihood ratio test (BLRT) (53). The lower the AIC, BIC and aBIC are, the better the model data fit is (61). The entropy value is an important indicator for evaluating the classification accuracy, ranging from 0 to 1, and an entropy value >0.80 indicates high separation among profiles (51). At the same time, significant LMR-LRT and BLRT tests indicate that the corresponding *k* class model is better than the *k*-1 class model (51). Secondly, the classification results of latent profile were taken as dependent variables, and gender was taken as independent variables to establish a multinomial logistic regression model. Finally, SPSS 23.0 was used for variance analysis to explore the impact of latent classes of positive solitude on positive and negative affect.

## Results

### Descriptive Results

The mean value, the standard deviation of each variable and the correlation matrix among variables are shown in Table 2. As the results showed, the association between gender and total score of positive solitude was not significant (*r* = 0.01, *p* > 0.05), but the difference was significant in subsequent latent profile analysis results. And positive solitude was significantly positively correlated with positive affect (*r* = 0.52, *p* < 0.001) and was negatively correlated with negative affect (*r*= −0.11, *p* < 0.01).

**Table 2:** Descriptive statistics and correlation analysis of each variable.

	***M***	***SD***	**1**	**2**	**3**	**4**
1. Gender^a^	–	–	1			
2. Positive solitude	3.98	0.54	0.01	1		
3. Positive affect	3.60	0.70	−0.11**	0.52***	1	
4. Negative affect	2.52	0.93	−0.002	−0.11**	−0.001	1

^a^Gender as a dummy variable, male = 0, female = 1; N = 902, ^**^p <0.01, ^***^p <0.001.

### LPA Results

The fit statistics of profile models are presented in Table 3. According to Entropy, the five-class model fits best. Moreover, the AIC, BIC and aBIC of the five-class model were the smallest, so the five-class model was selected as the final model.

**Table 3:** The results of latent profile analysis of positive solitude.

**Model**	**AIC**	**BIC**	**aBIC**	**Entropy**	**LMR (*p*)**	**BLRT (*p*)**	**Proportion of categories (%)**
2	17,574.770	17,709.300	17,620.376	0.809	<0.001	<0.001	45.90/54.10
3	17,103.333	17,285.909	17,165.227	0.822	<0.001	<0.001	14.30/51.66/34.04
4	16,995.184	17,225.805	17,073.365	0.768	<0.001	<0.001	40.69/37.25/11.53/10.53
5	16,346.580	16,625.247	16,441.049	0.882	<0.001	<0.001	17.74/23.61/14.41/25.94/18.29
6	16,862.427	17,189.140	16,973.183	0.823	<0.01	<0.001	0.11/10.75/40.02/1.55/35.92/11.64

*N = 902*.

**Figure 1:**
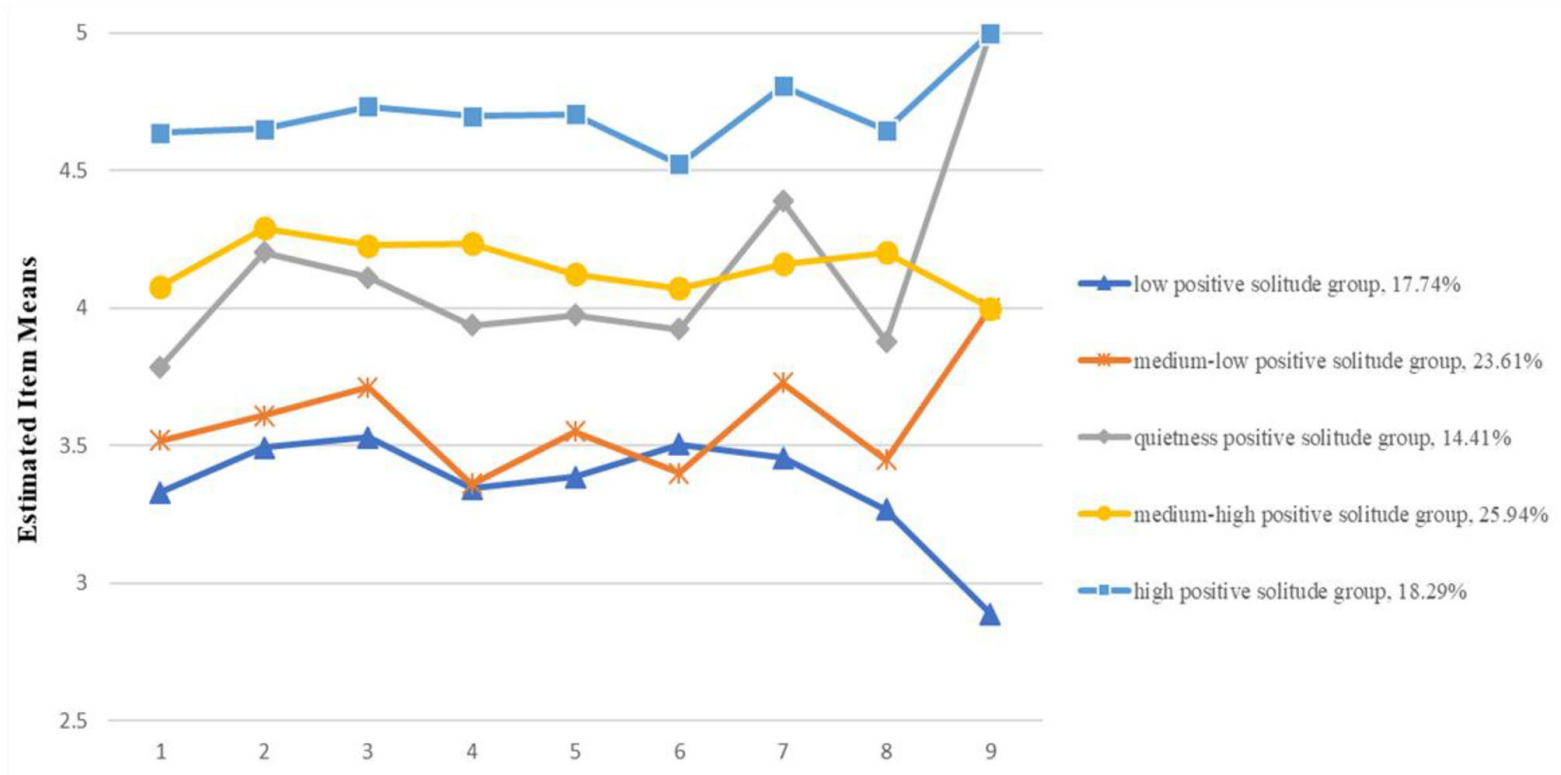
The five profiles of positive solitude by latent profile analysis.

Figure 1 depicts the score distribution of the latent class of positive solitude on all items. Class 1 consisted of 17.74% (*n* = 160) of adults who showed low positive solitude (labeled as the low positive solitude group). Class 2 included 23.61% (*n* = 213) of adults and was labeled as the medium-low positive solitude group because they showed medium-low levels of positive solitude. Class 3 consisted of 14.41% (*n* = 130) of adults who scored highest on item 9, which fell into the quietness category in the original initial pool (labeled as the quietness positive solitude group). Class 4 included 25.94% (*n* = 234) of adults who showed medium-low levels of positive solitude (labeled as the medium-low positive solitude group). Finally, Class 5 consisted of 18.29% (*n* = 165) of adults who showed high positive solitude in all items (labeled as the high positive solitude group).

### Associations Between Gender and Positive Solitude Profiles

The results of the multinomial logistic regression model were shown in Table 4. Females were more likely to be positive solitude than males in Profile 2 (Medium-low; β = 0.559, *SE* = 0.280, *p* < 0.05, *OR* = 1.749) and Profile 4 (Medium-high; β = 0.479, *SE* = 0.211, *p* < 0.05, *OR* = 1.614) in reference to Profile 3 (Quietness).

**Table 4:** Results of multinomial logistic regression analysis on gender.

**Reference profile**		**β**	***S.E*.**	***Est./S.E*.**	***P***	***OR***
Profile 1: Low						
	Profile 2: Medium-low	0.279	0.744	0.374	0.708	1.322
	Profile 3: Quietness	−0.280	0.717	−0.390	0.696	0.756
	Profile 4: Medium-high	0.199	0.712	0.280	0.779	1.220
	Profile 5: High	0.121	0.734	0.165	0.869	1.129
Profile 2: Medium-low						
	Profile 1: Low	−0.279	0.744	−0.374	0.708	0.757
	Profile 3: Quietness	−0.559	0.280	−1.995	0.046	0.572
	Profile 4: Medium-high	−0.079	0.278	−0.284	0.776	0.924
	Profile 5: High	−0.157	0.331	−0.476	0.634	0.855
Profile 3: Quietness						
	Profile 1: Low	0.280	0.717	0.390	0.696	1.323
	Profile 2: Medium-low	0.559	0.280	1.995	0.046	1.749
	Profile 4: Medium-high	0.479	0.211	2.269	0.023	1.614
	Profile 5: High	0.401	0.257	1.562	0.118	1.493
Profile 4: Medium-high						
	Profile 1: Low	−0.199	0.712	−0.280	0.779	0.820
	Profile 2: Medium-low	0.079	0.278	0.284	0.776	1.082
	Profile 3: Quietness	−0.479	0.211	−2.269	0.023	0.619
	Profile 5: High	−0.078	0.295	−0.265	0.791	0.925

*N = 902*.

### Differences Between Positive Solitude Profiles and Affect

**Figure 2:**
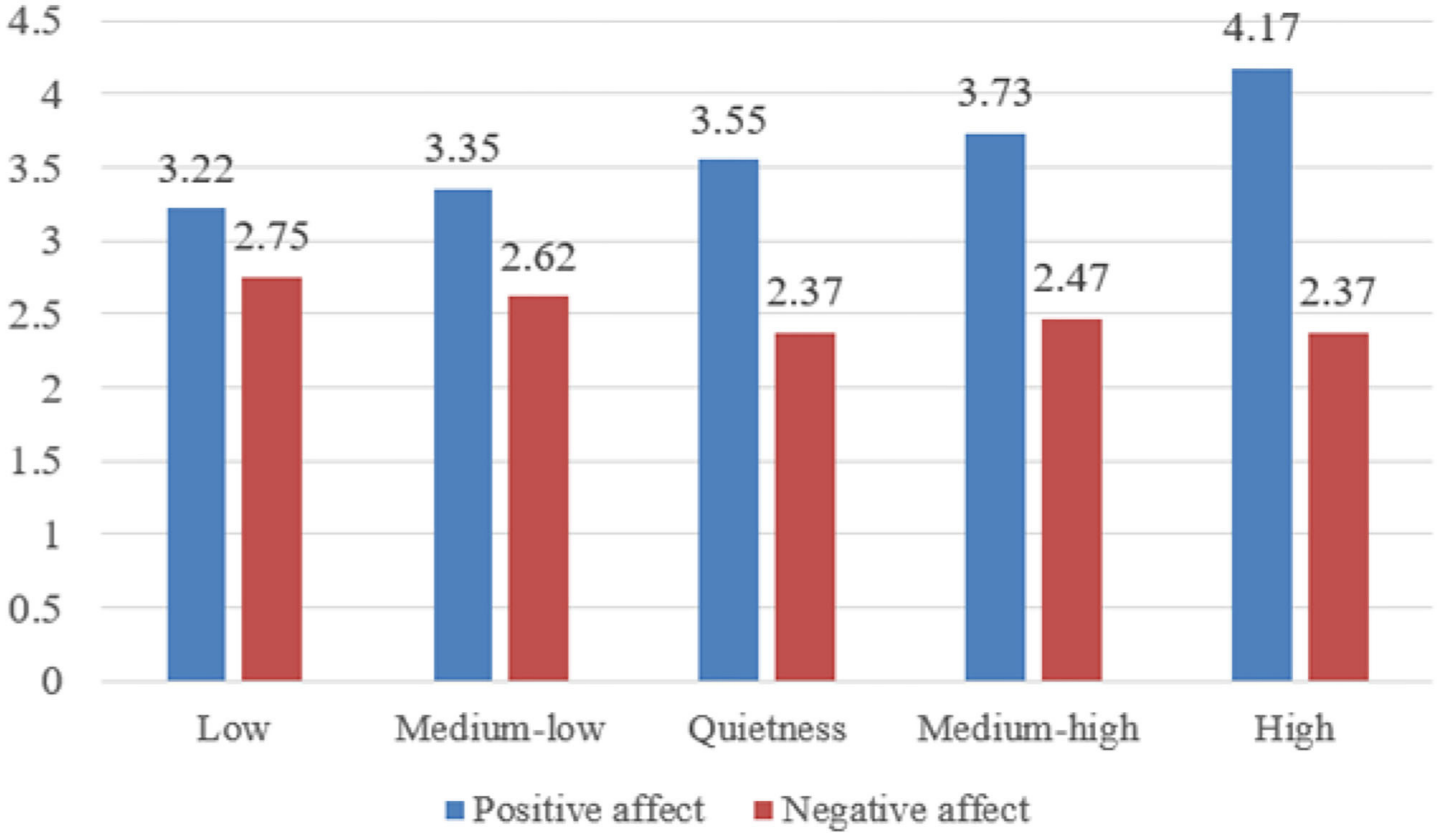
Bar graph for ANOVA.

The results are shown in Table 5. And the bar graph for ANOVA is shown in Figure 2. The mean level of positive affect was significantly different among the five groups [*F*_(4, 897)_ = 60.147, *p* < 0.001]. Specifically, the high positive solitude group had higher positive affect than the other four groups (*ps* < 0.001). The medium-high positive solitude group had higher positive affect than the low and the medium-low positive solitude group (*ps* < 0.001). The quietness positive solitude group had higher positive affect than the low (*p* < 0.05) and the medium-low positive solitude group (*p* < 0.001). However, there was no significant difference between the low and the medium-low positive solitude groups, and also between the quietness and the medium-high positive solitude groups.

**Table 5:** Differences in positive and negative affect across latent profiles (*M* ± *SD*).

	**Low**	**Medium-low**	**Quietness**	**Medium-high**	**High**	***F***	**η^2^**	***Post-hoc***
Positive affect	3.22 ± 0.59	3.35 ± 0.55	3.55 ± 0.67	3.73 ± 0.62	4.17 ± 0.72	60.147***	0.211	5 > 3 > 1; 5 > 4 > 1; 3 > 2; 4 > 2; 5 > 2
Negative affect	2.75 ± 0.78	2.62 ± 0.85	2.37 ± 0.89	2.47 ± 0.93	2.37 ± 1.12	5.347***	0.023	1 > 3; 1 > 4; 1 > 5

N = 902, ^***^p <0.001.

The mean level of negative affect was significantly different among the five groups [*F*_(4, 897)_ = 5.347, *p* < 0.001]. The low positive solitude group had higher negative affect than the quietness (*p* < 0.01), medium-high (*p* < 0.05) and high (*p* < 0.01) positive solitude groups. However, the medium-low positive solitude group did not differ from the other four groups. The quietness positive solitude group did not differ from the medium-high and the high positive solitude groups. And the differences between the medium-high and the high positive solitude groups were not statistically significant.

## Discussion

### Profiles of Positive Solitude in Chinese Adults

This study aimed to identify distinct profiles of positive solitude. Additionally, the current study used multinomial logistic regression to examine how these profiles related to gender. We also examined the differences in positive and negative affect across different latent profiles. Results showed five latent classes: low, medium-low, quietness, medium-high, and high positive solitude groups. Specifically, adults in the high positive solitude group scored highest on all items, indicating that they had high positive solitude and were better able to adapt to self-isolation during the recurrent outbreak of COVID-19. In contrast, adults in the low positive solitude group displayed low scores across all items, indicating that adults in this group were at a relative disadvantage in positive solitude ability and were more likely to have difficulty in adapting to isolation during the recurrent outbreak of COVID-19. In addition, the quality of life of adults in the quietness positive solitude group improved through positive solitude. According to Ost Mor's definition of “quiet positive solitude,” sometimes people need solitude to obtain a quiet environment or condition in order to calm down from their daily burdens. Everyone has a need for quiet, and solitude can help individuals obtain the conditions for quiet (27). Through solitude, individuals can escape from the society's hustle and bustle, let go of all defenses and pretensions, and truly enjoy their quiet time.

### Profiles' Association With Gender

Our findings found that gender was not significantly associated with the total score of positive solitude, but there were significant differences in gender across profiles. This is precisely the characteristic of the latent profile analysis: the overall association between gender and the total score of positive solitude is not significant because the traditional variable-centered approach ignores intra-individual differences, but when we explore the different profiles separately, we can find more detailed differential results. The results of the latent profile analysis partially support our hypothesis. In previous studies, the effects of gender on solitude were controversial, and however, as previously mentioned, the reason for such controversy was probably that those studies used the variable-centered approach. Although this variable-centered method was simple and effective, some critical individual differences were relatively ignored, which may lead to different conclusions. Multinomial logistic regression results for different models of positive solitude showed that compared with males, females were more likely to be positive solitude than males in Profile 2 (Medium-low) and Profile 4 (Medium-high) in reference to Profile 3 (Quietness). The results of this study suggested that during the recurrent outbreak of COVID-19, attention should be paid to the positive solitude of individuals and whether they also suffered from the difficulty of positive solitude during the isolation period, especially for males. The study found that the positive solitude of males was not as good as that of females, which suggested that a heavier emphasis needs to be placed on males' positive solitude.

### Association Between Profiles and Affect

Our hypothesis about the association between profiles and positive and negative affect is basically supported. We found that the positive affect of the high positive solitude group was higher than the other four groups, and the low positive solitude group had higher negative affect than the quietness, medium-high and high positive solitude groups. This suggested that positive solitude was accompanied by more positive affect and less negative affect. It demonstrated that positive solitude played a key role in mood regulation. Based on the results of positive and negative affect of Chinese adults during the recurrent outbreak of COVID-19, we can provide supportive care to individuals at risk of emotional distress. And in practical work, we should attach great importance to the positive solitude of individuals with high negative affect.

### Limitations and Future Directions

There are some limitations to the current survey which we need to note. First of all, our research hypothesis assumed that gender differences would have an impact on positive solitude, and the results showed that gender causes differences in positive solitude in different profiles. However, the analysis in this study is not sufficient for the differential impact of gender, and more research should be conducted to examine the gender difference in future studies. Secondly, all variables were assessed by self-report measurement in our study, which may affect the validity of this study. Since response bias and social desirability effects may have influenced the results. In the future, measures with less social desirability effect can be taken into consideration. Thirdly, due to the large sample size, detailed information on the social background of the sample was not available for this study. In future studies, detailed background information of the subjects should be investigated. Finally, given the differences between individualistic and collectivistic cultures and the fact that COVID-19 is currently spreading in many countries worldwide, there may be a need for further investigation and validation of positive solitude affected by COVID-19 in some other diverse samples from different countries.

## Conclusion

This study found five latent profiles of positive solitude (low positive solitude group, medium-low positive solitude group, quietness positive solitude group, medium-high positive solitude group and high positive solitude group) among Chinese adults. The results of the multinomial logistic regression model showed that females were more likely to display positive solitude than males in Profile 2 and Profile 4 in reference to Profile 3 (Quietness). These profiles had a unique pattern of association with both positive and negative emotions. The high positive solitude group had higher positive affect than the other four groups; The low positive solitude group had higher negative affect than the quietness, medium-high and high positive solitude groups.

## Data Availability Statement

The raw data supporting the conclusions of this article will be made available by the authors, without undue reservation.

## Author Contributions

ZY, BY, and YH designed the study, analyzed the data, and conceptualized the models. ZY collected the data. QY supervised the project. All authors have seen and approved the manuscript and wrote and revised the manuscript.

## Funding

This study was supported by the National Natural Science Foundation of China (72164018), National Social Science Fund Project (BFA200065), Jiangxi Social Science Foundation Project (21JY13), Jiangxi' Key Research Base Project of Humanities and Social Sciences (JD20068), and Science and Technology Research Project of Jiangxi' Department of Education (GJJ200306).

## Conflict of Interest

The authors declare that the research was conducted in the absence of any commercial or financial relationships that could be construed as a potential conflict of interest.

## Publisher's Note

All claims expressed in this article are solely those of the authors and do not necessarily represent those of their affiliated organizations, or those of the publisher, the editors and the reviewers. Any product that may be evaluated in this article, or claim that may be made by its manufacturer, is not guaranteed or endorsed by the publisher.
